# *SCN5A* Variants as Genetic Arrhythmias Triggers for Familial Bileaflet Mitral Valve Prolapse

**DOI:** 10.3390/ijms232214447

**Published:** 2022-11-21

**Authors:** Hager Jaouadi, Alexis Théron, Jérôme Hourdain, Hélène Martel, Karine Nguyen, Raja Habachi, Jean-Claude Deharo, Frédéric Collart, Jean-François Avierinos, Stéphane Zaffran

**Affiliations:** 1 Marseille Medical Genetics, INSERM, Aix Marseille Université, U1251 Marseille, France; 2 Department of Cardiac Surgery, La Timone Hospital, 13005 Marseille, France; 3 Department of Cardiology, La Timone Hospital, 13005 Marseille, France; 4 Department of Medical Genetics, Timone Enfant Hospital, 13005 Marseille, France

**Keywords:** familial mitral valve prolapse, genetic arrhythmia substrate, *SCN5A* mutation, H558R polymorphism, syncope, resuscitated sudden cardiac death

## Abstract

Mitral valve prolapse (MVP) is a common valvular heart defect with variable outcomes. Several studies reported MVP as an underestimated cause of life-threatening arrhythmias and sudden cardiac death (SCD), mostly in young adult women. Herein, we report a clinical and genetic investigation of a family with bileaflet MVP and a history of syncopes and resuscitated sudden cardiac death. Using family based whole exome sequencing, we identified two missense variants in the *SCN5A* gene. A rare variant *SCN5A*:p.Ala572Asp and the well-known functional *SCN5A*:p.His558Arg polymorphism. Both variants are shared between the mother and her daughter with a history of resuscitated SCD and syncopes, respectively. The second daughter with prodromal MVP as well as her healthy father and sister carried only the *SCN5A*:p.His558Arg polymorphism. Our study is highly suggestive of the contribution of *SCN5A* mutations as the potential genetic cause of the electric instability leading to ventricular arrhythmias in familial MVP cases with syncope and/or SCD history.

## 1. Introduction

Mitral valve prolapse (MVP) is a common valvulopathy with variable outcomes. It is characterized by fibromyxomatous changes in the mitral leaflet tissue and a systolic displacement of one or both mitral valve leaflets into the left atrium [1,2]. The estimated prevalence of MVP is between 1% and 3% in the general population [3,4]. The diagnosis is based on transthoracic echocardiography as a systolic billowing of one or both leaflets exceeding 2 mm beyond the mitral annulus in a long-axis view [1,5].

The complications spectrum of MVP is ranging from arrhythmias, stroke, endocarditis, mitral regurgitation (MR) requiring surgery, and sudden cardiac death (SCD) [6,7]. Arrhythmic MVP has been associated with sudden death with an incidence of up to 2% per year [8].

Recently, the association of MVP with ventricular arrhythmias as a substrate of SCD has been documented in patients with MVP. The major arrhythmogenic risk predictors reported include a history of syncope, ventricular ectopic beats (right bundle branch morphology), ventricular repolarization abnormalities, left ventricle fibrosis in the papillary muscles, and mitral annular disjunction, as well as a possible pro-arrhythmic genetic substrate [7,8,9].

A malignant subset of MVP has been correlated with an increased risk of sudden death [10]. This subtype is characterized by a bileaflet MVP occurring in young female patients with biphasic or inverted T waves in the inferior leads, and frequent complex ventricular ectopic activity with documented ventricular bigeminy or VT and premature ventricular contraction configurations of outflow tract alternating with papillary muscle or fascicular origin [9,10].

A strong hereditary component has been described by reporting several pedigrees for MVP [11,12,13]. Disse et al. identified four pedigrees suggesting an autosomal dominant pattern of inheritance. Genetic linkage analysis of the largest pedigree with 24 members in three generations showed a significant linkage for markers mapping to chromosome 16 (chr16p11.2-p12.1; MVP1-OMIM# 157700) [14]. Thereafter, missense mutations in the *DCHS1* gene have been identified in different families and segregated with the disease in affected members (MVP2-Locus MIM# 603057) [12,15]. Toomer et al. restudied a large family reported by Nesta et al. using exome sequencing and identified missense mutation in the *DZIP1* gene (MVP3-Locus MIM#608671). Thereafter, additional *DZIP1* variants have been prioritized in 42 MVP probands [16,17].

A recessive X-linked pattern of inheritance has been reported as well [18]. Indeed, missense mutations in the *FLNA* gene have been identified in MVP familial cases [19]. Delling et al. reported a four- to five-fold risk in the offspring of parents with MVP compared to parents without MVP, which highlight the relevance of genetic testing [1,11].

Here, we report the clinical and genetic investigation of a family with bileaflet myxomatous MVP complicated by several episodes of syncopes and a resuscitated SCD.

## 2. Results

### 2.1. Clinical Findings

The index case, a 42-year-old woman, was diagnosed with a bileaflet myxomatous MVP after a resuscitated SCD at the age 27 years old while she was in a church.

An exhaustive initial assessment was performed, and MVP was the only abnormality found to explain the occurrence of life-threatening ventricular arrhythmia.

**Figure 1:**
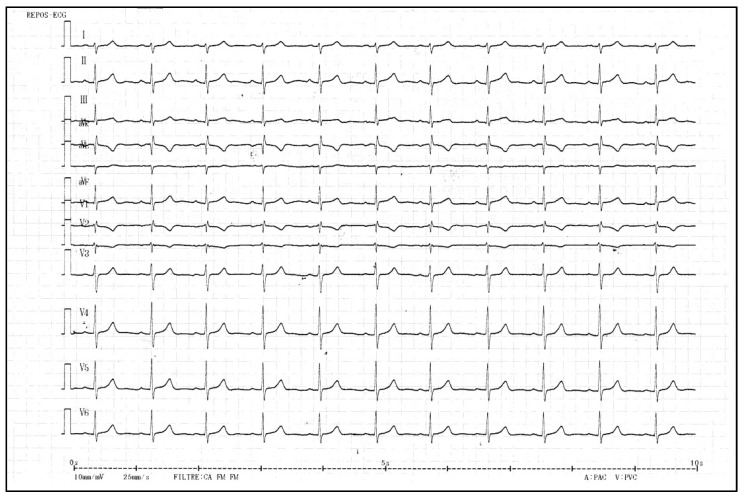
The 12 leads ECG at rest of the index case: a normal T wave inversion is present in the precordial leads V1 and V2 (QTc = 432 ms).

Her 12 leads ECG at rest showed a T wave inversion in the precordial leads V1 and V2. The corrected QT interval was normal (Figure 1). Therefore, she underwent internal cardioverter defibrillator implantation. Monitoring of the device revealed appropriate shocks on ventricular arrhythmia’s recidivism.

Of note, before defibrillator implantation, cardiac Magnetic Resonance Imaging (MRI) was performed and ruled out structural cardiac diseases such as arrhythmogenic right ventricular cardiomyopathy or hypertrophic/dilated cardiomyopathies. Cardiac MRI confirmed bileaflet MVP with myxomatous impairment (Barlow’s disease) reported at the first trans-thoracic echocardiography but without late Gadolinium enhancement. The patient also underwent coronary angiography with a methylergometrine provocation test and ventriculography. No lesions of coronary arteries, vasospasm, or takotsubo cardiomyopathy were noted. Finally, programmed ventricular stimulation with isoprenaline infusion and a provocation test with flecainide injection did not induce ventricular arrhythmia or unmasked an electrical aspect of Brugada syndrome on 12 leads surface ECG.

**Figure 2:**
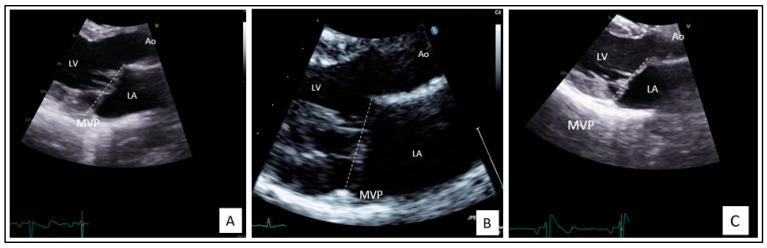
Echocardiography of the index case (**A**), the affected daughter (II-2) (**B**), and the oldest daughter with a prodromal MVP (II-1) (**C**). LV: left ventricle; LA: left atrium; Ao: aorta; MVP: mitral valve prolapse.

Her echocardiography follow-up showed a constant moderate MR, with mild myxomatous thickened leaflets, the presence of a posterior annulus disjunction, and a typical atrialization of posterior leaflet insertion and posterior systolic curling of the inferolateral wall of the left ventricular (LV) (Figure 2A). The left ventricular ejection fraction (LVEF) remains normal but the LV and left atrium (LA) are mildly dilated according to the European Society of Cardiology Guidelines [20,21].

Regarding the treatment, in the years preceding the cardiac arrest event, the patient consulted a cardiologist for palpitations, describing episodes of tachycardia with sudden onset and termination which have never been documented. The 12 leads ECG tracing, the trans-thoracic echocardiography, and the biology were reported as normal. A trial treatment with a calcium blocker (verapamil) was temporarily taken by the patient with the only hypothesis of paroxysmal junctional tachycardia. At discharge after cardiac arrest, the calcium blockers (diltiazem) were continued for 10 months. When the patient was appropriately treated (one electric shock) by the defibrillator for ventricular tachycardia (202 beats/min) a treatment with sotalol was initiated.

Family screening revealed one symptomatic daughter (Subject II-2) with a history of two syncopes at 11 years old while she was on a thrill ride and palpitations during an intense run. Her 12 leads ECG at rest, the ECG 24 h Holter, and the exercise stress test were normal. However, her echocardiography showed a bileaflet MVP which predominates on the anterior leaflet with mild myxomatous thickened leaflets, posterior annulus disjunction, and winding movement (Figure 2B). The MVP is complicated by a moderate MR. LVEF and LA volumes were normal. LV was mildly dilated. She received an implantable loop recorder for VA screening.

The oldest sister, 23 years old (Subject II-1), is currently asymptomatic. Her echocardiography revealed a prodromal MVP with a mild holosystolic MR, a posterior annulus disjunction, and a winding movement (Figure 2C). She had no cardiac remodeling and no systolic dysfunction.

The father (Subject I-1) had normal echocardiography.

### 2.2. Genetic Findings

Whole-exome sequencing analysis allowed us to prioritize two missense variants in the *SCN5A* gene. One heterozygous rare variant shared between the index case and her daughter (II-2) NM_000335:exon12:c.1715G>T; p.(Ala572Asp) (MAF = 0.0051 in the gnomAD database; MAF = 0.0032 in the European non-Finnish population). The variant is located in the DI–DII interdomain linker of Nav1.5 channel. The *SCN5A*:p.(Ala572Asp) is predicted as probably pathogenic by UMD-predictor and has a CADD-phred score = 19.44 (GRCh37-v1.6).

**Figure 3:**
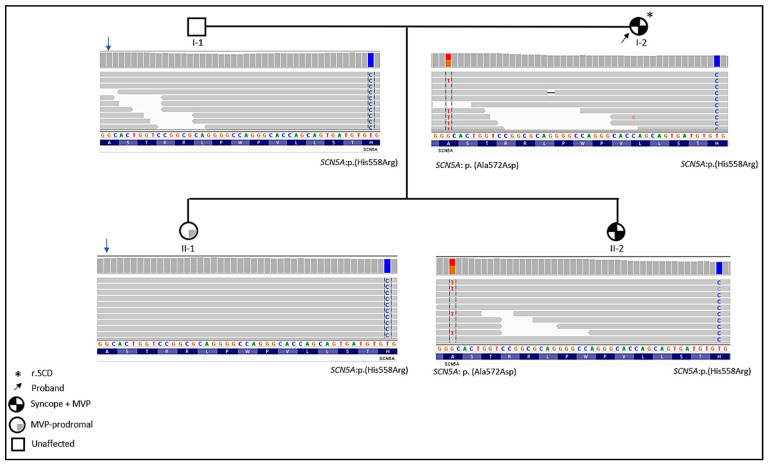
Family pedigree and integrative genomics viewer visualization showing the p.(Ala572Asp) and p.(His558Arg) in the *SCN5A* gene.

The second variant, NM_000335:exon12:c.1673T>C; p.(His558Arg) is found in all the family members. Thus, the father (I-1) and the oldest daughter (II-1) with prodromal MVP carried only the modulatory *SCN5A*:p.(His558Arg) polymorphism (Figure 3).

This common variant (MAF = 0.223) is extensively studied and well-known as a functional modifying polymorphism. Indeed, the p.(His558Arg) may act in a mutation-specific manner as a risk-allele or a protector allele in the presence of other rare and/or causal highly penetrant variants in the same gene or in separate gene [22,23,24,25].

Due to the close physical genetic distance between the Ala572 and His558, both alleles are in the “cis” phase. Thus, Ala572 and His558 are invariably linked. This “cis” configuration has been confirmed by Tester et al. [24]. Of note, in our cases, the His558Arg polymorphism was found in a homozygous state in all family members.

It is noteworthy that no other clinically relevant variants that may explain the phenotype in this family were found. More specifically, genes implicated in MVP, valvulopathies, and cardiac channelopathies were targeted and screened.

Both symptomatic family members (I-2 and II-2) carried the two variants. The father and the paucisymptomatic daughter (II-1) carried only the p.(His558Arg) polymorphism (Figure 3).

**Figure 4:**
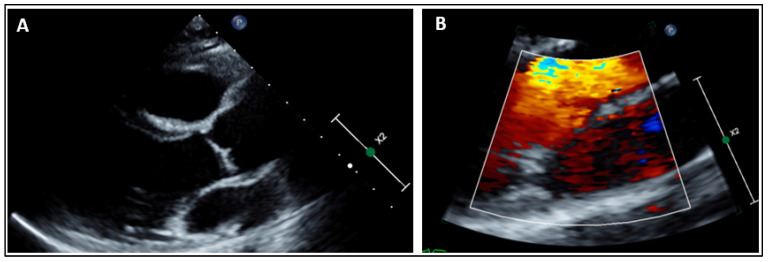
Parasternal long axis view ruling out any mitral valve prolapse (**A**). Mitral valve leaflets are thin without chordae elongation. Parasternal long axis view with color Doppler ruling out any mitral regurgitation in systole (**B**).

**Figure 5:**
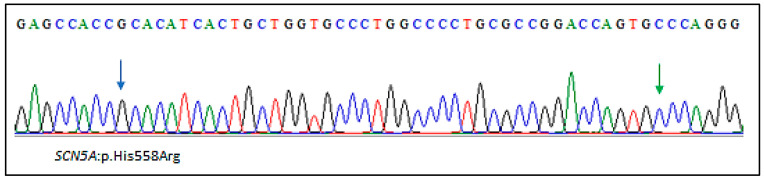
Sanger sequencing electropherogram showing the *SCN5A*:p.(His558Arg) polymorphism. The wild-type p.Ala572 residue is indicated by a green arrow.

More recently, another daughter of the family has been recruited for clinical and genetic screening. Her cardiac evaluation showed no MVP (Figure 4). Sanger sequencing revealed the absence of the *SCN5A*:p.(Ala572Asp) variant. She carried only the common *SCN5A*:p.(His558Arg) polymorphism (Figure 5).

## 3. Discussion

We report a family with bileaflet MVP female cases and a history of resuscitated SCD and syncope episodes. Whole exome sequencing of the family allowed us to identify a rare heterozygous variant *SCN5A*:p.(Ala572Asp) in the index case and her symptomatic daughter and the common polymorphism p.(His558Arg) in the same gene.

The *SCN5A* gene encodes the α-subunit of the voltage-gated sodium channel (Nav1.5), a key regulator of the inward sodium current. Mutations in the *SCN5A* gene cause different cardiac channelopathies with diverse phenotypes such as familial atrial fibrillation type 10 (OMIM#614022), Brugada syndrome (OMIM#601144), and long QT syndrome type 3 (OMIM#603830) [26,27,28]. *SCN5A* mutations have been also identified in patients with primary cardiomyopathies [26,29]. In addition to the pleiotropic nature of the *SCN5A* gene, intrafamilial phenotypic variability has been recently reported. Indeed, Balla et al. identified an *SCN5A* variant (p.Leu135Pro) in several family members with multiple cardiac diseases ranging from Brugada syndrome to arrhythmogenic cardiomyopathy [30].

The association of myxomatous bilealflet MVP and the *SCN5A* gene has been reported twice. Missov et al. identified a mutation in the *SCN5A* gene in a female MVP patient with out-of-hospital cardiac arrest and flail posterior leaflet leading to a severe eccentric MR [30]. The second reported patient by Mahajan et al. is a young adult woman as well (37 years old) with history of sudden cardiac arrest during a dance class. Two missense variants have been identified in this patient in *SCN5A* and *LMNA* genes [31]. Of note, our index case is 42 years old and her daughter (subject II-2) presented her first arrhythmic event at the age of 11 years old.

The *SCN5A*:p.(Ala572Asp) found in the present family has been studied by Tester et al. in order to assess its pathogenicity and contribution to the physiopathology of Long QT Syndrome (LQTS) [24]. Functional studies showed no gating kinetic or current density differences compared with wild-type channels when Asp572 was studied in the background of His558. However, when expressed with Arg558, significant kinetics changes were found [24]. Moreover, a threefold increase in late/persistent sodium current and slower time constants of recovery from inactivation were observed [24]. The authors have concluded that the presence of the p.His558Arg polymorphism along with the p.Ala572Asp variant produced Nav1.5 channels with moderate to altered LQT3-like gain-of-function properties [24].

Furthermore, the *SCN5A*:p.Ala572Asp mutation has been found in approximately 3% of European torsades de pointes–positive congestive heart failure or myocardial infarction cases compared to 0.7% of European controls [32,33]. More interestingly, the same mutation has been identified in 1.6% of female SCD cases compared with 0.27% of controls [32,34]. Thus, *SCN5A*:p.Ala572Asp seemed to be overrepresented in female proarrhythmic cases.

Ventricular and supraventricular arrhythmias were associated with complications of MVP [1,8,35]. Indeed, about 66% of MVP patients have a higher prevalence of ventricular arrhythmias (34%) with premature ventricular contractions as the most common pattern [35,36]. In addition, early repolarization and QT dispersion have been documented in MVP cases and suggested as arrhythmogenic factors [2,37].

Hourdain et al. reported the largest cohort of resuscitated patients with SCD in whom MVP was the only detectable cause [9]. MR has been demonstrated to be an independent arrhythmogenic risk factor. Nevertheless, polymorphic ventricular arrhythmias originating from the posterior papillary muscle with typical prolonged duration were reported as frequent and represented the main ventricular arrhythmia triggers. Long-term follow-up revealed the recurrence of life-threatening arrhythmias in those patients [9].

In the present family study, WES data were analyzed with a primary focus on genes related to MVP such as *DCHS1* and *DZIP1* but also on genes implicated in valvular and aortic defects. The only relevant variants found are *SCN5A*:p.(Ala572Asp) and *SCN5A*:p.(His558Arg).

In light of these findings, we hypothesized that the genetic substrate of arrhythmic MVP leading to life-threatening arrhythmias could be attributed to variants in the cardiac sodium channel.

In conclusion, MVP is an underestimated cause of arrhythmic SCD, mostly in young adult women. Genetic testing may play a crucial role to help stratify patients for more personalized clinical management and to identify at-risk family members early.

## 4. Materials and Methods

This study was conducted according to the principles of the Declaration of Helsinki and to the ethical standards of the first author’s institutional review board (Registration number: 2016-A00958-53).

### 4.1. Whole Exome Sequencing (WES)

Peripheral blood sample was collected after obtaining written informed consent from all family members included in this study or their guardians. Genomic DNA was extracted by standard techniques.

Family based WES was performed using the NimbleGen SeqCap EZ MedExome kit according to the manufacturer’s protocol (Roche Sequencing Solutions, Madison, MI, USA). Paired-end 150 bp reads from the DNA libraries were sequenced using Illumina NextSeq 500 platform (Illumina, San Diego, CA, USA). Raw fastQ files were aligned to the hg19 reference human genome (University of California Santa Cruz, UCSC) using the maximum exact matches algorithm in BWA software. Alignment quality was evaluated using Qualimap 2.2.1.

Variant calling and annotation were performed using GATK and ANNOVAR best practices, respectively. Variant annotation and exome analysis were performed with VarAFT software, version 2.17 (http://varaft.eu, accessed on 15 July 2022).

### 4.2. In Silico Analysis Tools

#### Variant Prioritization

Variant prioritization was carried out using Variant Annotation and Filtering Tool (VarAFT http://varaft.eu/, accessed on 15 July 2022). A pedigree-based analysis was performed considering autosomal dominance and recessivity as well as X-linked dominance and recessivity patterns of inheritance.

A pedigree-based analysis was performed considering both “common disease-rare variant” and “common disease-common variant” hypotheses. Our strategy was driven by the high prevalence of the MVP in the general population. Thus, we removed non-coding and synonymous variants, and no frequency filter was applied.

## Data Availability

The authors confirm that the data supporting the findings of this study are available within the article.
